# LINC01783 accelerated tongue squamous cell carcinoma progression via inhibiting miR‐199b‐5p

**DOI:** 10.1111/jcmm.16352

**Published:** 2021-08-07

**Authors:** Ying Hu, Xiaofeng Wang, Chong Li, Liang Jiao, Yi Du

**Affiliations:** ^1^ Jinan Stomatological Hospital Jinan China

**Keywords:** LINC01783, lncRNAs, miR‐199b‐5p, tongue squamous cell carcinoma

## Abstract

Growing studies illustrated that lncRNAs exert critical roles in development and occurrence of tumours including TSCC. In this research, we indicated that LINC01783 was up‐regulated in TSCC cells (SCC1, Cal27, UM1 and SCC4) when compared to NHOK cell. RT‐qPCR analysis indicated that LINC01783 was overexpressed in 22 TSCC cases (73.3%, 22/30) compared with no‐tumour specimens. LINC01783 level was up‐regulated in TSCC specimens when compared to no‐tumour specimens. Ectopic expression of LINC01783 promoted TSCC cell cycle and growth and EMT progression in both TSCC cell SCC1 and Cal27. Overexpression of LINC01783 sponged miR‐199b‐5p in TSCC cell and elevated expression of LINC01783 inhibited miR‐199b‐5p expression. Moreover, we illustrated that miR‐199b‐5p was down‐regulated in TSCC cells and specimen and LINC01783 level was up‐regulated in TSCC specimens when compared to no‐tumour specimens. Elevated expression of LINC01783 promoted TSCC cell growth, cycle and EMT progression by sponging miR‐199b‐5p. These data suggested that LINC01783 functioned as one oncogene and might be one treatment target for TSCC.

## 1 INTRODUCTION

SCC (squamous cell carcinoma) represents the 10th most common solid tumour worldwide, and TSCC (tongue squamous cell carcinoma) is the most frequent type of SCC.^1^, ^2^, ^3^, ^4^ TSCC usually caused to malfunction of speech, deglutition and mastication.^5^, ^6^, ^7^, ^8^ TSCC was famous for its high proportion of proliferation and metastasis of lymph nodes.^9^, ^10^, ^11^ Although attempts such as chemotherapy and surgery have been tried, the five‐year survival rate of TSCC remains unsatisfactory.^3^, ^5^ Thus, it is better to explore new biomarkers and targets for TSCC.

lncRNAs (>200 nucleotides in length) originally considered as the transcriptional noise and were emerging as novel modulators in cancer paradigm.^12^, ^13^, ^14^, ^15^, ^16^ Growing evidence illustrated that lncRNAs regulate abundant disease development including tumour, neuropathic pain, intervertebral disc degeneration, Hirschsprung's disease, tuberculosis and atherosclerosis.^17^, ^18^, ^19^, ^20^, ^21^, ^22^, ^23^ lncRNAs modulated cell processes such as fate decision, proliferation, metabolism, immunity and differentiation.^24^, ^25^, ^26^, ^27^, ^28^ Recently, a study noted that a novel lncRNA LINC01783 has been revealed to be up‐regulated in cervical cancer and LINC01783 overexpression enhanced cervical cancer cell migration, invasion and growth and inhibited cell apoptosis.^29^ Increasing studies demonstrated that lncRNAs played important roles in TSCC development. The cell functions and underlying mechanisms of LINC01783 in TSCC are still unclear.

Our data illustrated that LINC01783 was up‐regulated in TSCC cells and specimen and ectopic expression of LINC01783 promoted TSCC cell cycle and growth and EMT progression.

## 2 MATERIALS AND METHODS

### 2.1 Clinical specimens and cell Culture and Transfection

TSCC specimens and paracancerous specimens were obtained from Liaocheng people's Hospital, and case samples were collected with written informed consent. Our research was approved with Ethics Board of Jinan Stomatological Hospital due to Declaration of Helsinki. NHOK cell and TSCC human cells (SCC1, Cal27, UM1 and SCC4) were obtained from the Chinese Academy of Sciences of Shanghai. Cells were seeded in DMEM supplemented with antibiotic and FBS. Mimic of miR‐199b‐5p and scramble, pcDNA‐LINC01783 and control vectors were composed from GenePharma and transfected to cells with Lipofectamine 2000.

### 2.2 RT‐qPCR

RNA of cell or specimen was isolated using TRIzol reagent. RNA quantity was assessed with spectrophotometer. RT‐qPCR assay was utilized to assess miR‐199b‐5p and LINC01783 level with SYBR Green PCR Mix on the Bio‐Rad CFX PCR System. U6 or GAPDH was utilized as reference control for miRNA and LINC01783, respectively. Method of 2^−DΔΔCt^ was used to determine gene level. The primers are as follows: for LINC01783, forward: 5′‐CAAGG ACAGC AGGTG GAGTA‐3′, reverse: 5′‐CTTAC AGTGG ACTCG GGGTT‐3′; GAPDH, forward: 5′‐GGGAGCCAAAAGGGTCAT‐3′, reverse: 5′‐GAGTCCTTCCACGATACCAA‐3′; and U6, forward: 5′‐CTCGCTTCGGCAGCACA‐3′, reverse: 5′‐ AACGCTTCACGAATTTGCGT‐3′.

### 2.3 Cell proliferation and cycle assay

Cells were cultured in the 96‐well dishes at about density of 5000 cells/well and were detected at the 0, 1, 2 and 3 days. Each well was added with 10 μL CCK‐8 solution and incubated for 3 hours. The OD (optical density) was measured on spectrophotometer at 450 nm wavelength. Flow cytometry assay was utilized to study cell cycle. Cell was harvested and then washed in cold PBS and fixed by cold 70% ethanol overnight. Cell was incubated with PI (propidium iodide) solution on the ice for one half hour. Cell cycle was determined with flow cytometry (FACSC, BD).

### 2.4 Dual‐luciferase reporter assay

Wild‐type vectors of LINC01783 (WT‐LINC01783) and mutant‐type vectors of LINC01783 (mut‐LINC01783) were established. After culturing cells into 24‐well dish, WT‐LINC01783 or mut‐LINC01783 and miR‐199b‐5p mimic or scramble were contransfected by Lipofectamine 2000. After 2 days, luciferase intensities were determined with luciferase reporter reagent (Promega).

### 2.5 Statistical analysis

Results were evaluated by SPSS 19.0 software and shown as mean ± SD. Spearman's correlation assay was utilized to assess the correlation between LINC01783 and miR‐199b‐5p. *t* Test was constructed to determine significant difference between two groups. *P* < 0.05 was supposed to be statistically significant.

## 3 RESULTS

### 3.1 LINC01783, miR‐199b‐5p and DDR1 level in TSCC cells

**FIGURE 1:**
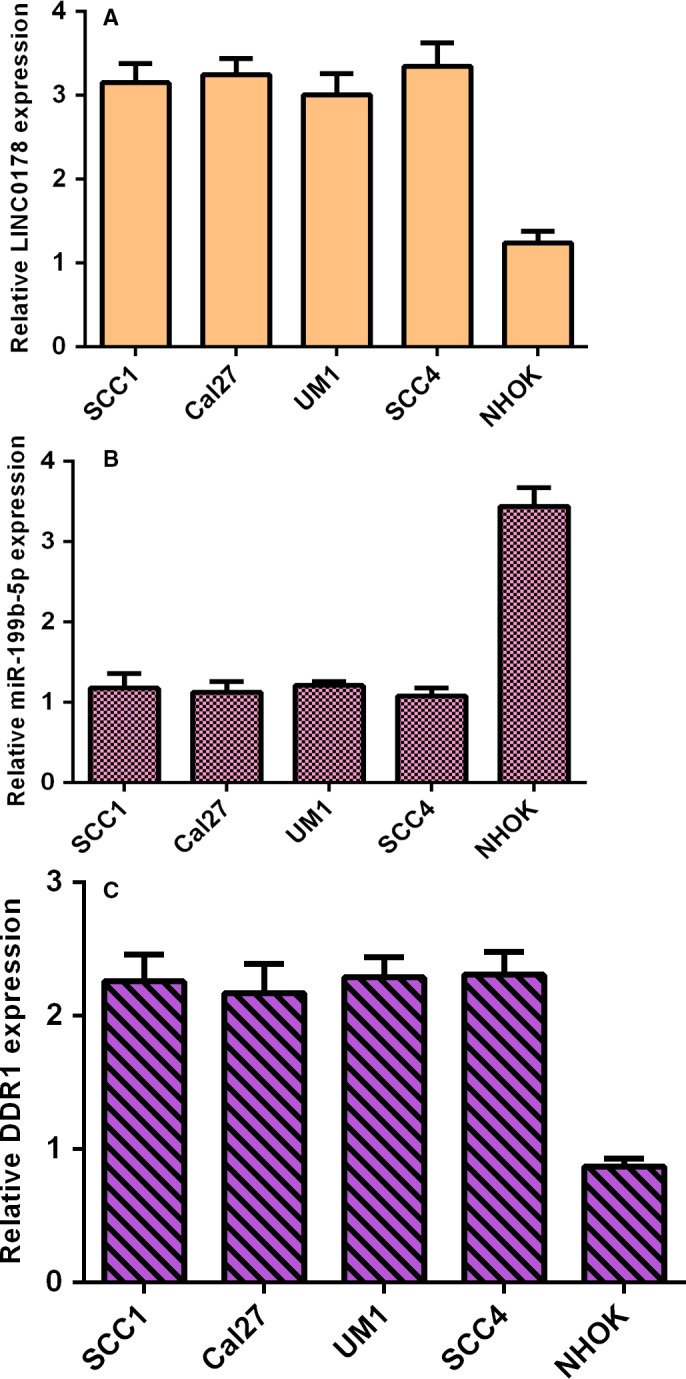
LINC01783, miR‐199b‐5p and DDR1 level in TSCC cells. (A) The expression of LINC01783 was detected by RT‐qPCR analysis. (B) miR‐199b‐5p was down‐regulated in TSCC human cells (SCC1, Cal27, UM1 and SCC4) when compared to NHOK cell. (C) The expression of DDR1 was detected by RT‐qPCR analysis

As Figure 1A showed, LINC01783 was overexpressed in TSCC human cells (SCC1, Cal27, UM1 and SCC4) when compared to NHOK cell. However, miR‐199b‐5p was down‐regulated in TSCC human cells (SCC1, Cal27, UM1 and SCC4) when compared to NHOK cell (Figure 1B). DDR1 was up‐regulated in TSCC human cells (SCC1, Cal27, UM1 and SCC4) when compared to NHOK cell (Figure 1C).

### 3.2 LINC01783 level in TSCC specimen

**FIGURE 2:**
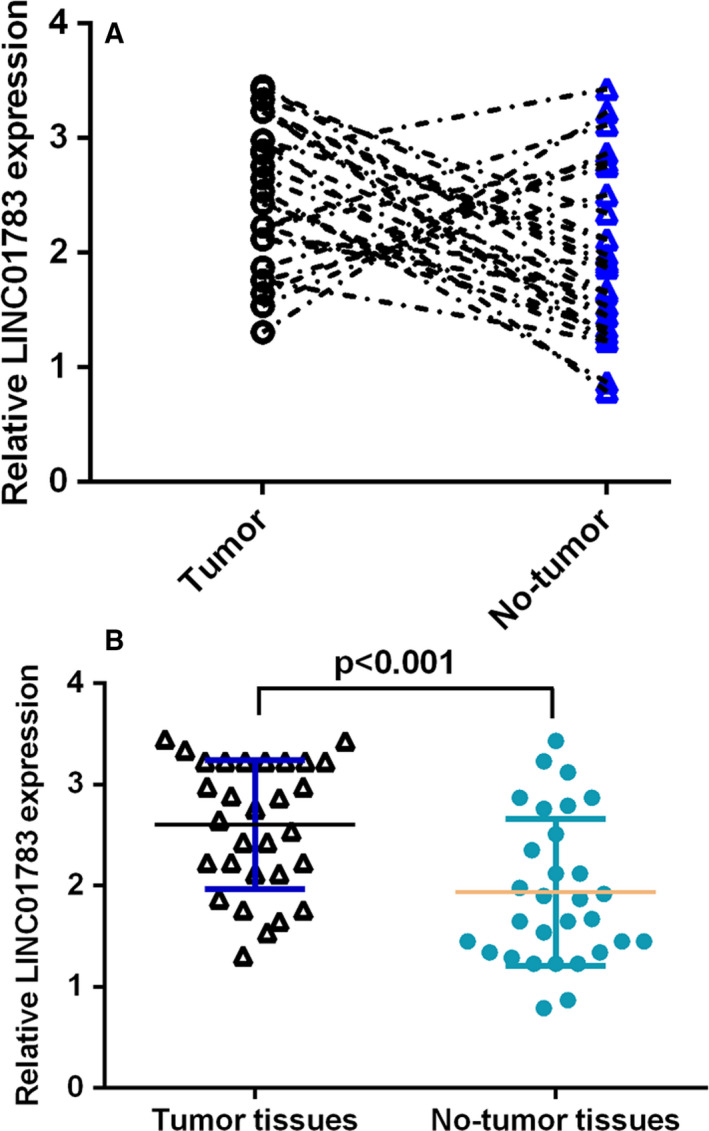
LINC01783 level in TSCC specimen. (A) RT‐qPCR analysis indicated that LINC01783 was overexpressed in 22 TSCC cases (73.3%, 22/30) compared with no‐tumour specimens. (B) LINC01783 level was up‐regulated in TSCC specimens when compared to no‐tumour specimens

Then, we studied the LINC01783 level in TSCC specimen. RT‐qPCR analysis indicated that LINC01783 was overexpressed in 22 TSCC cases (73.3%, 22/30) compared with no‐tumour specimens (Figure 2A). As Figure 2B indicated, LINC01783 level was up‐regulated in TSCC specimens when compared to no‐tumour specimens.

### 3.3 LINC01783 promoted TSCC cell cycle and growth

**FIGURE 3:**
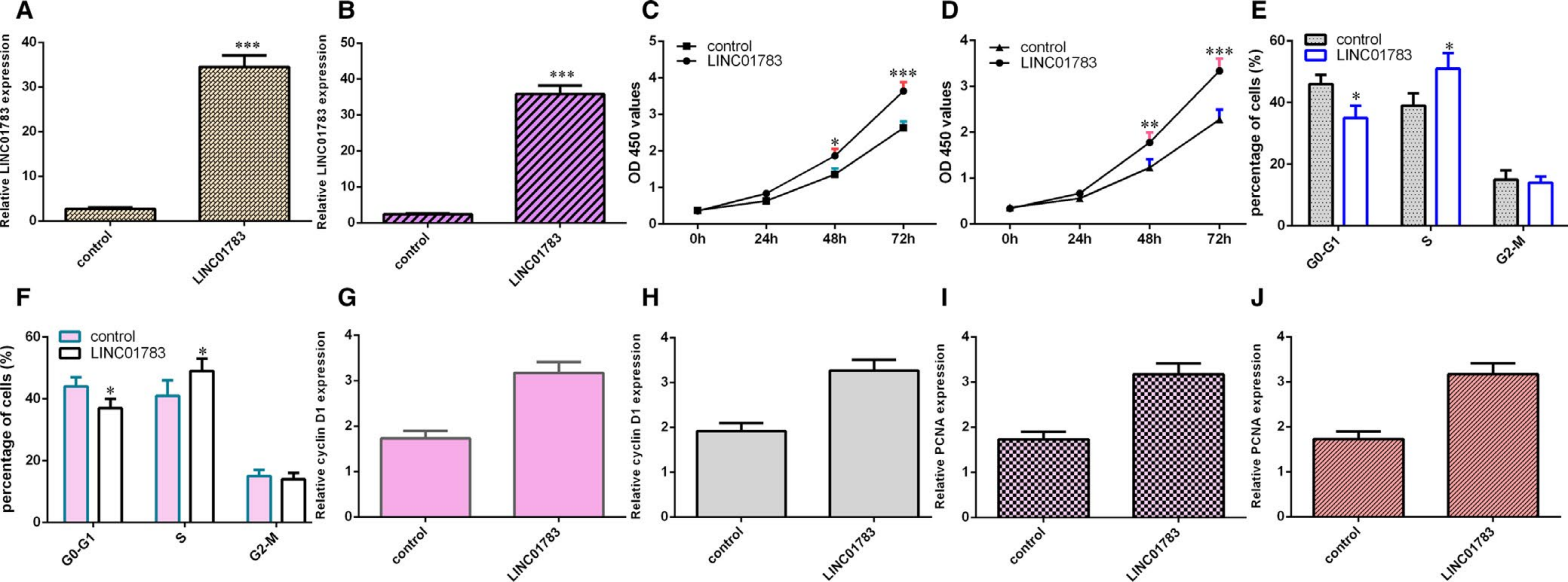
LINC01783 promoted TSCC cell cycle and growth. (A) The expression of LINC01783 was measured by RT‐qPCR analysis. (B) LINC01783 was overexpressed in Cal27 cell after transfected by pcDNA‐ LINC01783. (C) Ectopic expression of LINC01783 enhanced cell growth in SCC1. (D) Cell proliferation was detected by RT‐qPCR analysis. (E) Elevated expression of LINC01783 induced SCC1 cell cycle. (F) Overexpression of LINC01783 promoted Cal27 cell cycle. (G) The expression of cyclin D1 was detected through RT‐qPCR analysis. (H) The level of cyclin D1 was detected by RT‐qPCR analysis. (I) Overexpression of LINC01783 promoted PCNA expression in SCC1 cell. (J) The expression of PCNA was detected through RT‐qPCR analysis. *P < 0.05 and ***P < 0.001

We constructed pcDNA‐control and overexpression vector pcDNA‐LINC01783 and LINC01783 was overexpressed in SCC1 (Figure 3A) and Cal27 (Figure 3B) cell after transfected by pcDNA‐ LINC01783. Ectopic expression of LINC01783 enhanced cell growth in both SCC1 (Figure 3C) and Cal27 (Figure 3D) cell. Elevated expression of LINC01783 induced cell cycle both in SCC1 (Figure 3E) and in Cal27 (Figure 3F) cell. LINC01783 overexpression increased cyclin D1 expression both in SCC1 (Figure 3G) and in Cal27 (Figure 3H) cell. Overexpression of LINC01783 promoted PCNA expression both in SCC1 (Figure 3I) and Cal27 (Figure 3J) cell.

### 3.4 LINC*01783 induced EMT (epithelial to mesenchymal transition) progression*

**FIGURE 4:**
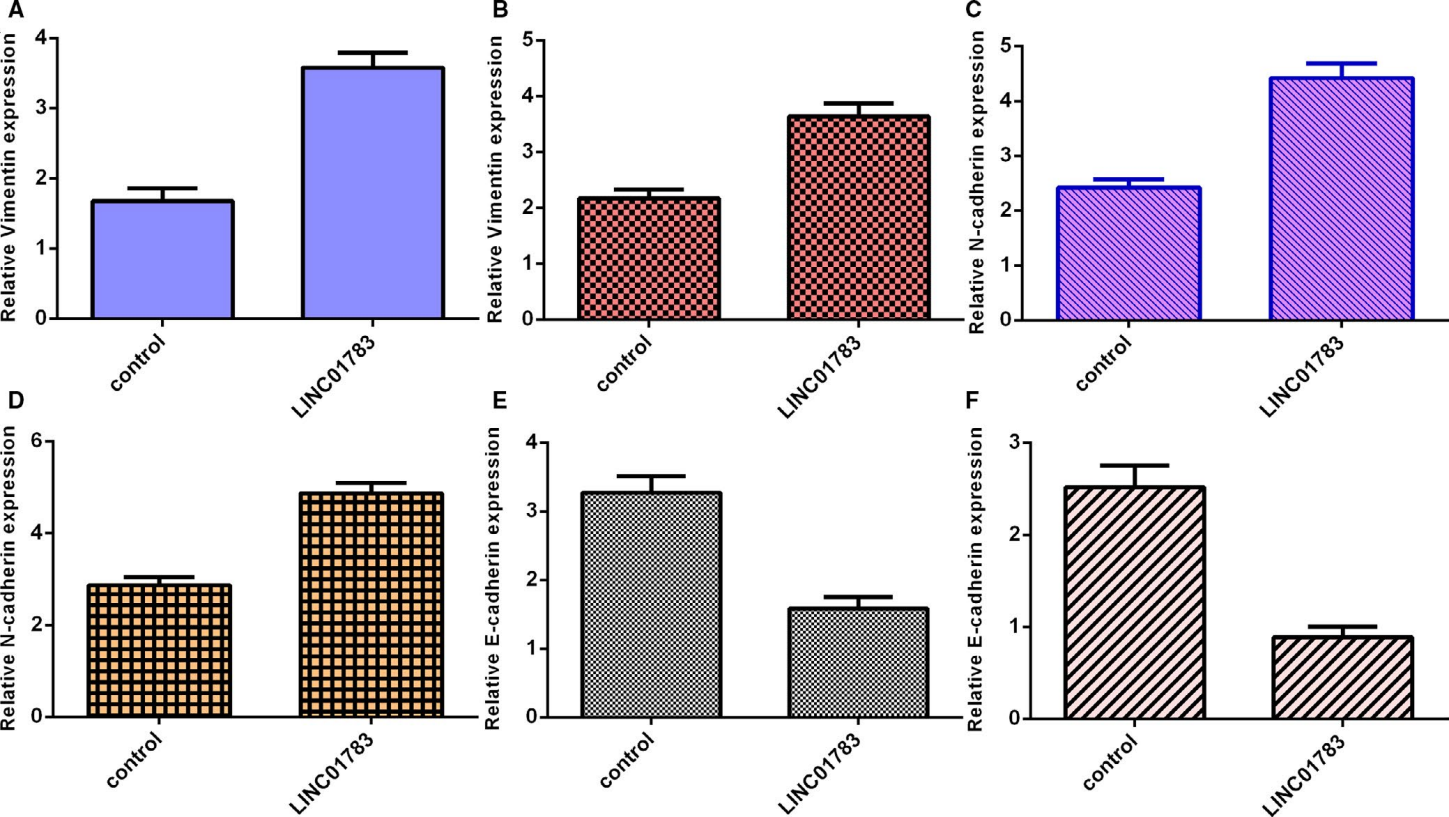
LINC01783 induced EMT (epithelial to mesenchymal transition) progression. (A) The expression of vimentin was measured by RT‐qPCR assay. (B) Ectopic expression of LINC01783 increased vimentin expression in Cal27 cell. (C) The expression of N‐cadherin was measured by RT‐qPCR assay. (D) Up‐regulation expression of LINC01783 promoted N‐cadherin expression in Cal27 cell. (E) The expression of E‐cadherin was measured by RT‐qPCR assay. (F) Overexpression of LINC01783 suppressed E‐cadherin expression in Cal27 cell

Ectopic expression of LINC01783 increased vimentin expression both in SCC1 (Figure 4A) and Cal27 (Figure 4B) cell. Up‐regulation expression of LINC01783 promoted N‐cadherin expression both in SCC1 (Figure 4C) and Cal27 (Figure 4D) cell. Furthermore, overexpression of LINC01783 inhibited E‐cadherin expression both in SCC1 (Figure 4E) and Cal27 (Figure 4F) cell.

### 3.5 LINC01783 sponged miR‐199b‐5p in TSCC cell

**FIGURE 5:**
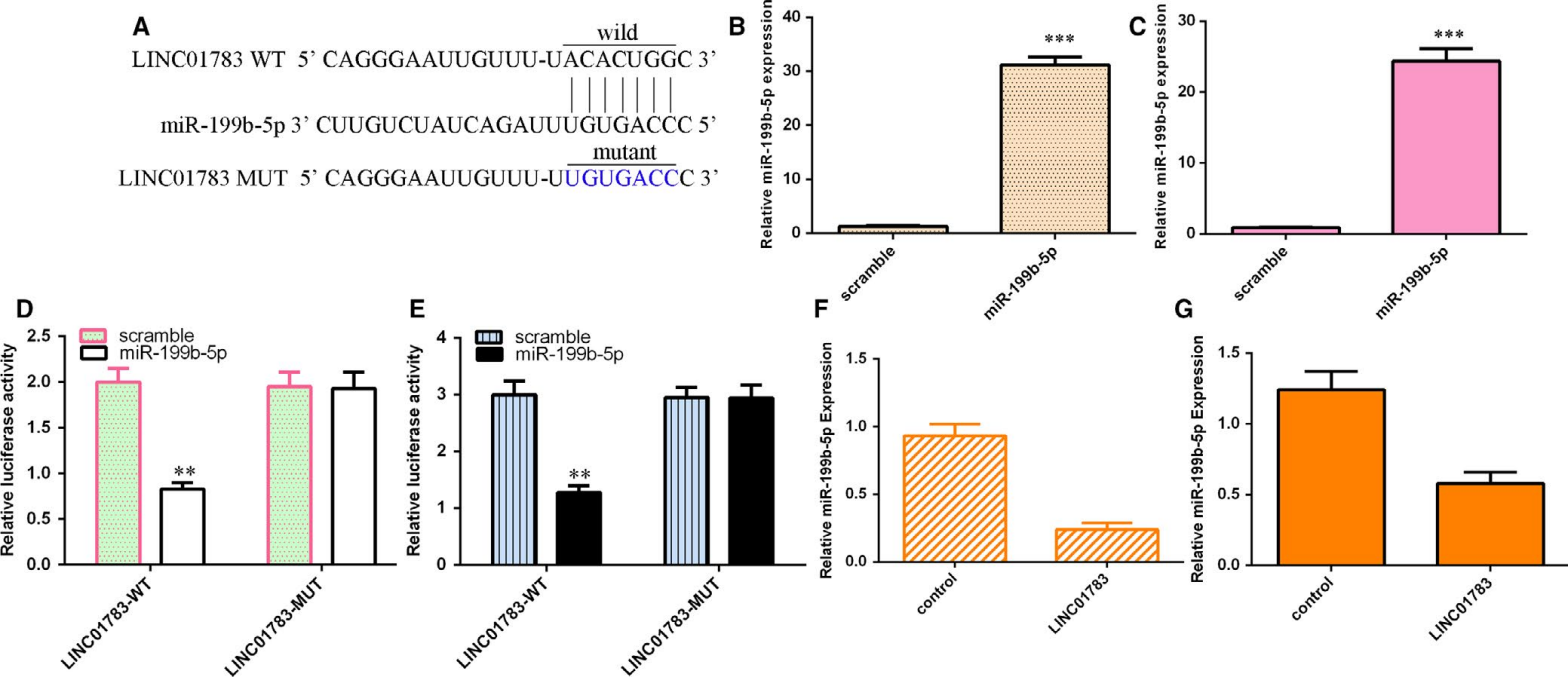
LINC01783 sponged miR‐199b‐5p in TSCC cell. (A) To learn mechanism by how LINC01783 modulated TSCC cell function, we predicted potential target miRNAs of LINC01783 using StarBase tool. (B) The expression of miR‐199b‐5p was determined by RT‐qPCR assay. (C) The expression of miR‐199b‐5p was determined using RT‐qPCR assay. (D) Luciferase reporter assay data illustrated that luciferase activity in SCC1 cell treated with LINC01783‐WT was decreased with treatment by miR‐199b‐5p mimic, whereas miR‐199b‐5p mimic had no effect on the luciferase activity in cell treated with LINC01783‐MUT. (E) The luciferase reporter assay data in Cal27 cell was shown. (F) Ectopic expression of LINC01783 inhibited miR‐199b‐5p expression in SCC1 cell. (G) The expression of miR‐199b‐5p was measured by RT‐qPCR assay. **P < 0.01 and ***P < 0.001

To learn mechanism by how LINC01783 modulated TSCC cell function, we predicted potential target miRNAs of LINC01783 using StarBase tool (http://starbase.sysu.edu.cn/index.php) (Figure 5A). The miR‐199b‐5p was significantly up‐regulated both in SCC1 (Figure 5B) and Cal27 (Figure 5C) cell after treated with miR‐199b‐5p mimic compared to scramble group. Luciferase reporter assay data illustrated that luciferase activity in SCC1 (Figure 5D) and Cal27 (Figure 5E) cell treated with LINC01783‐WT was decreased with treatment by miR‐199b‐5p mimic, whereas miR‐199b‐5p mimic had no effect on the luciferase activity in cell treated with LINC01783‐MUT. Ectopic expression of LINC01783 inhibited miR‐199b‐5p expression in both SCC1 (Figure 5F) and Cal27 (Figure 5G) cell.

### 3.6 miR‐199b‐5p level in TSCC specimen

**FIGURE 6:**
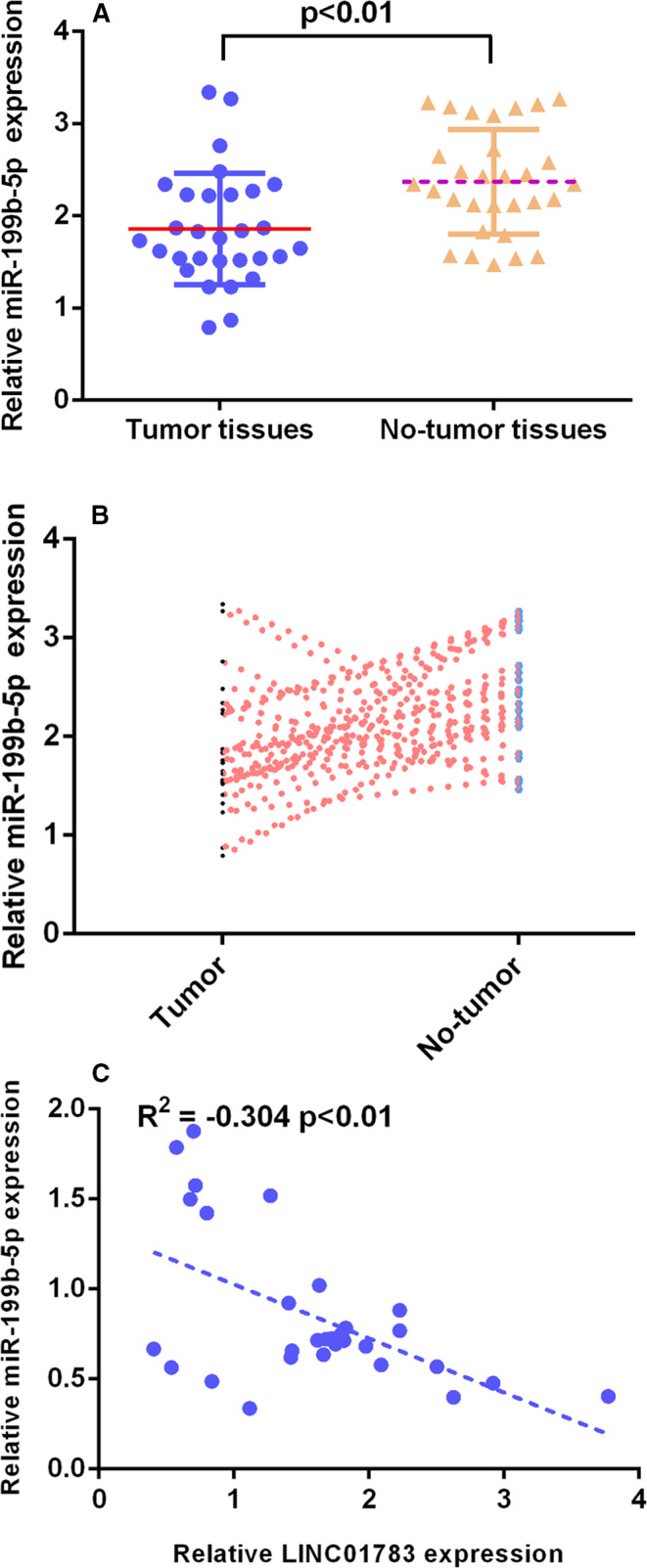
miR‐199b‐5p level in TSCC specimen. (A) RT‐qPCR analysis indicated that miR‐199b‐5p level was down‐regulated in TSCC specimens when compared to no‐tumour specimens. (B) miR‐199b‐5p was decreased in 22 TSCC cases (76.7%, 23/30) compared to no‐tumour specimens. (C) Pearson's assay indicated that miR‐199b‐5p was negatively associated with LINC01783 in TSCC specimens

Then, we studied the miR‐199b‐5p level in TSCC specimen. RT‐qPCR analysis indicated that miR‐199b‐5p level was down‐regulated in TSCC specimens when compared to no‐tumour specimens (Figure 6A). As Figure 6B indicated, miR‐199b‐5p was decreased in 22 TSCC cases (76.7%, 23/30) compared with no‐tumour specimens. Pearson's assay indicated that miR‐199b‐5p was negatively associated with LINC01783 in TSCC specimens (Figure 6C).

### 3.7 LINC01783 promoted TSCC cell growth and cycle by sponging miR‐199b‐5p

**FIGURE 7:**
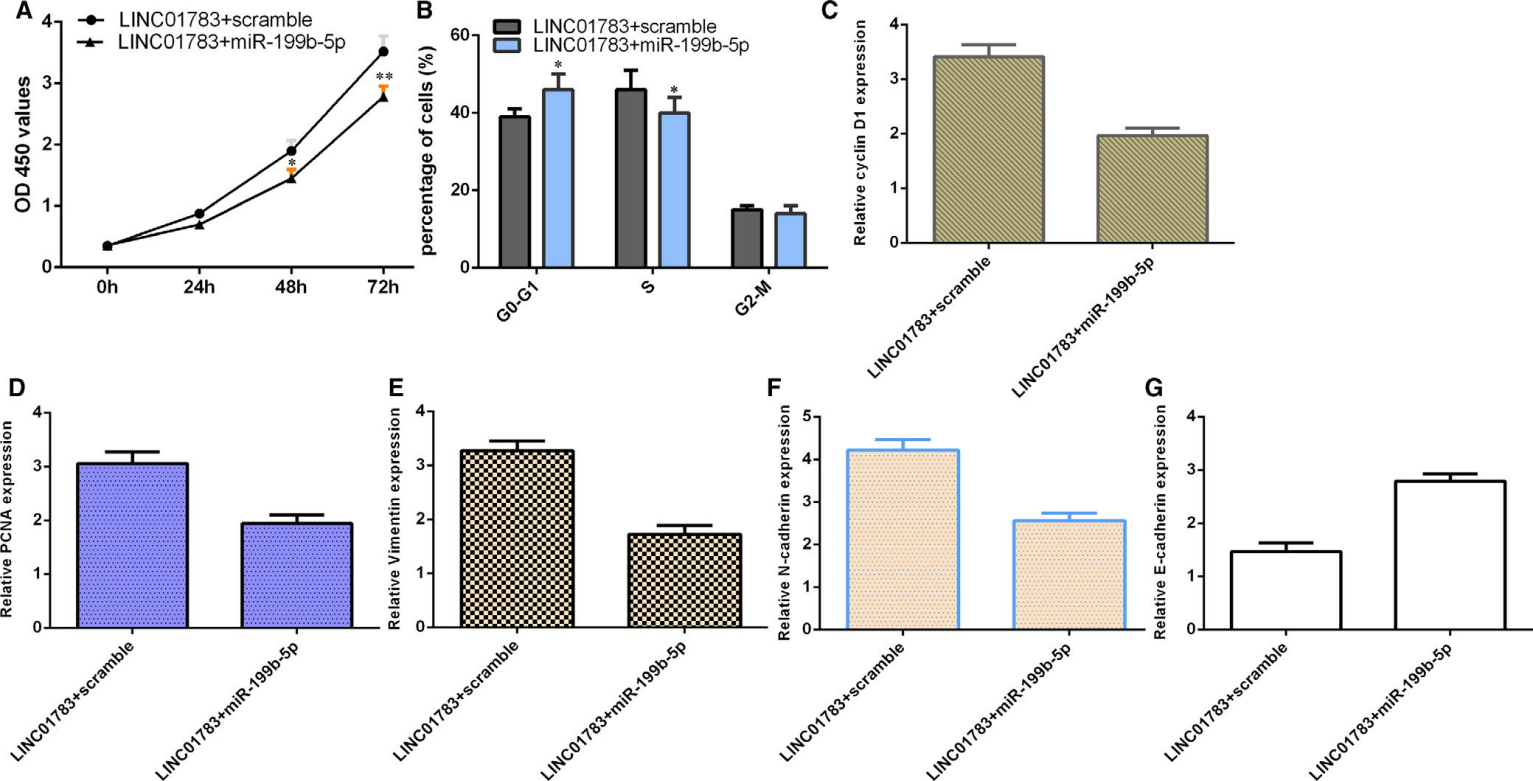
LINC01783 promoted TSCC cell growth and cycle by sponging miR‐199b‐5p. (A) The cell proliferation was measured by RT‐qPCR assay. (B) Overexpression of miR‐199b‐5p decreased cell cycle in LINC01783‐overexpressing SCC1 cell. (C) The expression of cyclin D1 was detected by RT‐qPCR analysis. (D) The expression of PCNA was measured by RT‐qPCR analysis. (E) The level of vimentin was detected using RT‐qPCR assay. (F) The expression of N‐cadherin was measured by RT‐qPCR analysis. (G) The expression of E‐cadherin was determined using RT‐qPCR assay. *P < 0.05, and **P < 0.01

To study whether LINC01783 promoted TSCC cell growth and cycle through sponging miR‐199b‐5p, we treated SCC1 cell with pcDNA‐LINC01783 and then transfected with miR‐199b‐5p mimic or scramble. Figure 7A and Figure 7B demonstrated that overexpression of miR‐199b‐5p decreased cell growth and cycle in LINC01783‐overexpressing SCC1 cell, respectively. Moreover, overexpression of miR‐199b‐5p suppressed cyclin D1 (Figure 7C) and PCNA (Figure 7D) expression in LINC01783‐overexpressing SCC1 cell. Elevated expression of miR‐199b‐5p inhibited vimentin (Figure 7E) and N‐cadherin (Figure 7F) and promoted E‐cadherin (Figure 7G) expression in LINC01783‐overexpressing SCC1 cell.

## 4 DISCUSSION

Growing studies have illustrated that lncRNAs exert critical roles in development and occurrence of tumours including TSCC.^28^, ^30^, ^31^, ^32^, ^33^ Our results suggested that LINC01783 was up‐regulated in TSCC cells and specimen and ectopic expression of LINC01783 promoted TSCC cell cycle and growth and EMT progression in both TSCC cell SCC1 and Cal27. Overexpression of LINC01783 sponged miR‐199b‐5p in TSCC cell and elevated expression of LINC01783 inhibited miR‐199b‐5p expression. Moreover, we illustrated that miR‐199b‐5p was down‐regulated in TSCC cells and specimen and LINC01783 level was up‐regulated in TSCC specimens when compared to no‐tumour specimens. Elevated expression of LINC01783 promoted TSCC cell growth, cycle and EMT progression by sponging miR‐199b‐5p. These data noted that LINC01783 functioned as one oncogene and might be one treatment target for TSCC.

Previous reference showed that CASC9 induced TSCC cell growth, invasion and migration via modulating miR‐423‐5p/SOX12.^34^ PART1 inhibited TSCC cell growth, migration and invasion through regulating miR‐503‐5p.^35^ Another study indicated that lncRNA ELDR increased cell growth through inducing cyclin E1‐ILF3 signalling in oral tumour.^36^ Xiong et al^37^ indicated that HOTTIP expression was elevated in TSCC cells and specimens and down‐regulated expression of HOTTIP suppressed TSCC cell migration, invasion and growth partly via regulating miR‐124‐3p/HMGA2. Zheng et al^38^ indicated that ectopic expression of DANCR increased TSCC cell migration, invasion and growth by sponging miR‐135a‐5p/KLF8. Recently, a study illustrated that a novel lncRNA LINC01783 has been revealed to be overexpressed in cervical cancer and LINC01783 overexpression enhanced cervical cancer cell migration, invasion and growth and inhibited cell apoptosis.^29^ The cell functions and underlying mechanisms of LINC01783 in TSCC are still unclear. We firstly studied the LINC01783 expression in TSCC cell. Our results indicated that LINC01783 was overexpressed in TSCC human cells (SCC1, Cal27, UM1 and SCC4) when compared to NHOK cell. RT‐qPCR analysis indicated that LINC01783 was overexpressed in 22 TSCC cases (73.3%, 22/30) compared with no‐tumour specimens. LINC01783 was up‐regulated in TSCC cells and specimen and ectopic expression of LINC01783 promoted TSCC cell cycle and growth and EMT progression in both TSCC cell SCC1 and Cal27. These data illustrated that LINC01783 functioned as one oncogene in TSCC.

Previous reference indicated that lncRNAs binded to miRNAs or other genes and modulated the expression of miRNAs for their therapeutic purposes.^39^, ^40^, ^41^, ^42^ For example, Jia et al^43^ showed that lnRNA FALEC suppressed TSCC metastasis and proliferation via inhibiting ECM1 by EZH2. Li et al^44^ demonstrated that lncRNA ADAMTS9‐AS2 induced TSCC growth, EMT and migration by sponging miR‐600/EZH2. Chen et al^45^ indicated that RP5‐916L7.2 enhanced TSCC cell growth and suppressed apoptosis via sponging miR‐939 and miR‐328. Yuan et al^46^ illustrated that down‐regulation of MALAT1 inhibited TSCC invasion, growth and migration via regulating PI3K/Akt/MMP‐9. Ren et al^47^ illustrated that lncRNA CRNDE induced TSCC cell invasion and proliferation by inhibiting miR‐384. Furthermore, LINC01783 promoted cervical cancer cell progression via sponging miR‐199b‐5p.^29^ To learn mechanism by how LINC01783 modulated TSCC cell function, we predicted potential target miRNAs of LINC01783 using StarBase 3.0. We found that LINC01783 may sponge miR‐199b‐5p and luciferase reporter assay data illustrated that luciferase activity in TSCC cell treated with LINC01783‐WT was decreased with treatment by miR‐199b‐5p mimic, whereas miR‐199b‐5p mimic had no effect on the luciferase activity in cell treated with LINC01783‐MUT. Elevated expression of LINC01783 inhibited miR‐199b‐5p expression. In addition, we illustrated that miR‐199b‐5p was down‐regulated in TSCC cells and specimen and LINC01783 level was up‐regulated in TSCC specimens when compared to no‐tumour specimens. Elevated expression of LINC01783 promoted TSCC cell growth, cycle and EMT progression by sponging miR‐199b‐5p.

Taken together, we illustrated that LINC01783 was up‐regulated in TSCC cells and specimen and ectopic expression of LINC01783‐induced TSCC cell cycle and growth and EMT progression via sponging miR‐199b‐5p.

## CONFLICT OF INTEREST

There is no conflict of Interest.

## AUTHOR CONTRIBUTION

**Ying Hu:** Conceptualization (equal); Data curation (equal); Formal analysis (equal); Investigation (equal); Writing‐original draft (equal); Writing‐review & editing (equal). **Xiaofeng Wang:** Conceptualization (equal); Data curation (equal); Formal analysis (equal); Resources (equal); Software (equal); Supervision (equal). **Chong Li:** Data curation (equal); Formal analysis (equal); Funding acquisition (equal); Project administration (equal); Supervision (equal); Validation (equal). **Liang Jiao:** Data curation (equal); Project administration (equal); Resources (equal); Software (equal); Supervision (equal). **Yi Du:** Conceptualization (lead); Data curation (lead); Formal analysis (equal); Funding acquisition (equal); Investigation (equal); Methodology (equal); Project administration (lead); Resources (equal); Software (equal); Supervision (equal); Validation (equal); Visualization (equal); Writing‐original draft (equal); Writing‐review & editing (equal).

## Data Availability

Research data are not shared.
